# Risk Assessment of Heavy Metals Occurrence in Two Wild Edible Oyster Mushrooms (*Pleurotus* spp.) Collected from Rajaji National Park

**DOI:** 10.3390/jof8101007

**Published:** 2022-09-25

**Authors:** Ivan Širić, Pankaj Kumar, Bashir Adelodun, Sami Abou Fayssal, Rakesh Kumar Bachheti, Archana Bachheti, Fidelis O. Ajibade, Vinod Kumar, Mostafa A. Taher, Ebrahem M. Eid

**Affiliations:** 1 University of Zagreb, Faculty of Agriculture, Svetosimunska 25, 10000 Zagreb, Croatia; 2 Agro-Ecology and Pollution Research Laboratory, Department of Zoology and Environmental Science, Gurukula Kangri (Deemed to Be University), Haridwar 249404, Uttarakhand, India; 3 Department of Agricultural and Biosystems Engineering, University of Ilorin, PMB 1515, Ilorin 240003, Nigeria; 4 Department of Agricultural Civil Engineering, Kyungpook National University, Daegu 37224, Korea; 5 Department of Agronomy, Faculty of Agronomy, University of Forestry, 10 Kliment Ohridski Blvd, 1797 Sofia, Bulgaria; 6 Department of Plant Production, Faculty of Agriculture, Lebanese University, Beirut 1302, Lebanon; 7 Department of Industrial Chemistry, College of Applied Science, Addis Ababa Science and Technology University, Addis Ababa P.O. Box 16417, Ethiopia; 8 Centre of Excellence in Nanotechnology, Addis Ababa Science and Technology University, Addis Ababa P.O. Box 16417, Ethiopia; 9 Department of Environmental Science, Graphic Era (Deemed to be University), Dehradun 248002, Uttarakhand, India; 10 Department of Civil and Environmental Engineering, Federal University of Technology, PMB 704, Akure 340001, Nigeria; 11 Biology Department, Faculty of Science and Arts, King Khalid University, Mohail Assir 61321, Saudi Arabia; 12 Botany Department, Faculty of Science, Aswan University, Aswan 81528, Egypt; 13 Biology Department, College of Science, King Khalid University, Abha 61321, Saudi Arabia; 14 Botany Department, Faculty of Science, Kafrelsheikh University, Kafr El-Sheikh 33516, Egypt

**Keywords:** food quality, forest biodiversity, health risk, metal elements, traditional foraging, wild mushrooms

## Abstract

This study aimed at assessing the concentration of six heavy metals (Cd, Cr, Cu, Fe, Mn, and Zn) in two wild edible oyster mushrooms (*Pleurotus ostreatus* and *Pleurotus djamor*) collected from Rajaji National Park in Haridwar, India. For this purpose, mushroom samples were collected from selected locations (forest, residential, tourist, industrial areas, and transportation activities) from June 2021 to July 2022 and subsequently analyzed for selected heavy metals using atomic absorption spectroscopy (AAS). Results showed that both *Pleurotus* spp. had significantly varying (*p* < 0.05) concentrations of heavy metals. However, *P. ostreatus* showed relatively higher concentration levels of these metals compared to *P. djamor*. The mean concentrations (mg/kg dry weight) of the Cd, Cr, Cu, Fe, Mn, and Zn in *P. ostreatus* and *P. djamor* were 0.10 and 0.08, 0.87 and 0.64, 16.19 and 14.77, 28.49 and 27.15, 9.93 and 8.73, and 18.15 and 15.76, respectively. As indicated by the multivariate analysis tools i.e., principal component analysis (PCA) and hierarchical cluster analysis (HCA), the locations near the residential, industrial, and transportation activities had higher concentration levels of heavy metals. Moreover, the health risk studies using the target hazard quotient (THQ < 1) showed no significant health risk as the consumption of both *Pleurotus* spp., except for at one location, had high-traffic activities. The findings of this study provide vital information about the occurrence of potentially toxic heavy metals in wild edible *Pleurotus* spp. in Rajaji National Park in Haridwar, India representing a safeguard for mushroom consumers.

## 1. Introduction

Mushrooms are protein-rich and low-fat sources of food, making them nutritionally equivalent to meat for the rising vegan community around the world [1]. According to a recent report, the global mushroom market accounted for 15.25 million tons in 2021 and is projected to reach 24.05 million tons by 2028 with a compound annual growth rate of 6.74% during the 2021–2028 period [2]. India accounts for around 2% of the world’s commercial mushroom production (around 0.31 million tons), however, several communities still depend on foraging wild mushrooms [3]. Being rich in vitamin D, riboflavin, and essential amino and fatty acids [4], mushroom consumption increased during the COVID-19 pandemic, showing positive impacts on human immunity. Whereas, only a little increase was observed in India as the consumption of mushrooms recently faced with a lack of appetite in some local areas due to its strangeness to traditional cuisine [5]. Despite this local behavior, this consumption is becoming trendier among youths due to the increasing awareness vis-à-vis its health benefits and low-cost production, as tremendous amounts of raw material based on agro-industrial wastes could be easily found [6,7].

Wild mushroom foraging is a cultural tradition of European countries, mainly in Central and Eastern Europe. Locals tend to forage collectively in the autumn–spring season, in which a diversity of delicious edible mushrooms flourish [3]. Yet, these knowledge and differentiation skills are not popular globally. For instance, some Indian locals tend to avoid wild mushroom foraging and consumption as they found it hard to differentiate between edible, non-edible, and poisonous species. Moreover, a lack of research on wild mushroom species in the Indian sub-continent constitutes an additional limiting factor for this exciting adventure [8]. Additional constraints of wild mushroom consumption include their growth in polluted zones near mines and factories. These sites are sources of potentially toxic heavy metals such as cadmium (Cd), cobalt (Co), copper (Cu), chromium (Cr), iron (Fe), lead (Pb), manganese (Mn), mercury (Hg), nickel (Ni), selenium (Se) and zinc (Zn). In this context, a recent report revealed a high potential of some wild edible mushroom species such as *Tricholoma* spp. to accumulate Cd concentrations with a bioconcentration factor (BCF) higher than 1 [9]. However, the authors noted no potential health risk, as the latter’s index (HRI) besides the daily intake of heavy metals was lower than 1 and 0.426, respectively. Although these results were very promising, the high bioaccumulation capacity is a matter of concern for researchers as many sources of pollution may be concealed in other locations. Earlier, wild *Agaricus campestris*, *Boletus edulis*, and *Boletus reticulatus* were found to be high accumulators of Cd and Hg, respectively [10]. Most of these potentially toxic heavy metals were detected in mushrooms’ caps rather than stipes. Being the most-desired part of mushrooms, cap accumulation of highly undesired elements is a worrying issue. Therefore, the need to study the environmental ecosystem (mainly soils) where wild edible mushrooms grow is always a needed fore step. It was reported that high levels of Cd were thought to be the main cause of breast, pancreas, and kidney cancers. Other potentially toxic heavy metals such as Hg can lead to the disruption of growth factor synthesis and affect reproduction, respectively [11]. Recently, the health risk and heavy-metal composition of *Agaricus bisporus* mushrooms sold in Uttarakhand State local markets were assessed [12]. Authors found a decreasing order of heavy metals as: Fe > Zn > Mn > Cr > Cu > Ni > Cd, with no potential hazard in consumption. However, further research should be performed on other local wild and edible species found in markets in order to assure safe mushroom consumption with no short or long-term health risks.

Oyster mushroom species (*Pleurotus* spp.) are mainly grown on lignocellulosic-rich materials or dead wood. These species are a great source of human protein, natural amino acids, and essential poly- and monounsaturated fatty acids [13,14]. Recently, it was acknowledged that *P. ostreatus* grown on agro-industrial residues such as olive pruning residues and spent coffee grounds had the potential to accumulate some desirable and undesirable metals. However, the reported concentrations were within the same limits set by the European Commission (EC), World Health Organization (WHO), and United States Food and Drug Administration (FDA) [15]. In the same vein, *P. ostreatus*, grown on spent mushroom substrate, supplemented with nano-amino additives outlined high Pb levels, (>0.3 mg/kg) exceeding limits set by the guidelines, which stipulate the need to reconsider consuming the produce regularly [14]. However, wild *P. ostreatus* mushrooms were acknowledged to enclose higher concentrations of heavy metals compared to cultivated ones. In this context, this species collected from mine-polluted soils delineated Zn, Cd, and Cr concentrations higher than the safe limits, with concentrations in stalks higher than those in caps [16]. Although the authors did not detect human carcinogenic risks, they simulated possible and serious health hazards if continuously consumed by locals. *P. djamor* is well appreciated for its high antioxidants and protein content. This species has been reported to accumulate the highest amounts of heavy metals among *Pleurotus* spp., and mainly has the highest Pb concentrations [17]. These authors also acknowledged the following decreasing order in terms of total metal bioaccumulation: *P. cirinopileatus* > *P. djamor* > *P. eryngii* > *P. ostreatus*.

Rajaji National Park is a famous natural reserve located on both sides of the Ganges River, Uttarakhand (820 km^2^). It is well known for its high biodiversity and is home to several rare plant and animal species [18]. This park is also a reserve for elephants and tigers, being the first park in Uttarakhand to offer the most prestigious wildlife conservation. Rajaji National Park hosts wild Safaris for tourists and locals all around the year [19]. In this context, wild mushroom populations settling in Rajaji National Park were very scantly investigated and clumsily identified in the literature [20]. Therefore, the current study investigates the heavy metal accumulation of two identified wild edible *Pleurotus* spp. and assesses their potential risk for human consumption. The findings of this study suggest a heavy-metal accumulation potential of wild edible *Pleurotus* spp. that could potentially be harmful to consumers.

## 2. Materials and Methods

### 2.1. Description of Study Area and Mushroom Sampling

**Figure 1:**
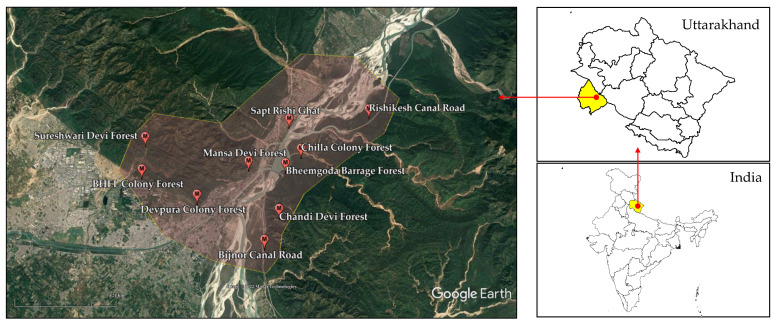
Map showing the sampling locations of *Pleurotus* spp. collection in and around Rajaji National Park at Haridwar, Uttarakhand, India (Source: Google Earth Pro).

For the present study, samples of two oyster mushroom species i.e., *P. ostreatus* and *P. djamor* were obtained from ten (10) locations around the Chilla Forest Range of Rajaji National Park in Haridwar city, Uttarakhand, India. Table 1 and Figure 1 show the list and map of sampling locations for the current study, respectively. The elevation of sampling locations ranged from 286–515 m. Moreover, the perimeter and total area of the sampling area were 42 km and 100 km^2^, respectively. The area receives an average annual rainfall of 2136.7 mm with August as the wettest month. The average temperature and humidity of the area are recorded as 23 °C and 68%, respectively [21]. The monsoon season in the region begins in July and lasts up to September. During this period, a wide variety of wild mushrooms appear in the forest areas, including *Pleurotus* spp. The sampling locations are easily accessible to the local communities and do not need any special permission from the forest authorities. Therefore, wild mushrooms are commonly collected and consumed by the local people. The mushroom samples were collected from June 2021 to July 2022 following standard field sampling methods [22]. The mushroom bodies were carefully picked and immediately transported to the laboratory for further analysis. However, the number of samples collected (*P. ostreatus*, *n* = 49; *P. djamor*, *n* = 39) for each species varied due to their availability. Herein, the *Pleurotus* spp. were morphologically identified using the field guide of Kumari et al. [23] and several online databases such as https://www.mushroom.world/ and http://www.mycokey.com/ (accessed on 1 September 2022).

### 2.2. Heavy-Metal Analysis

The collected mushroom samples were oven dried at 60 °C until a constant weight was achieved. The dried samples were again converted into fine powder form using a mechanical grinder (JMG3345, 450W, Usha, Gurgaon, India). Analysis of six heavy metals (Cd, Cr, Cu, Fe, Mn, Zn) was performed using atomic absorption spectroscopy (AAS, ECIL, Hyderabad, India). For this, 1 g of powdered mushroom sample was placed in a 150 mL-capacity conical flask and dissolved in 10 mL of a di-acid mixture having a 3:1 ratio of concentrated HNO_3_ and HClO_4_. The mixture was placed on a rotary shaker and self-digested for 12 h. After the completion of self-digestion, a 3% HNO_3_ solution was added to make the final volume of the contents 50 mL. Again, the flask was placed on a hot plate and digested for 1 h at 150 °C until 10 mL of residual volume was left. Finally, more HNO_3_ solution was added to make the liquid volume 50 mL and usable for heavy metal analysis using AAS. The analytical conditions of the AAS instrument such as detection limits, wavelength (nm), cathode lamp current (mA), slit width (0.2–0.7 mm), and flame (air-acetylene mixture) were adjusted based on the heavy metals as given in the previous study [24,25,26]. The standard recovery percentage of heavy metals ranged from 98.50–99.10%. All reagents used for heavy metal analysis were of laboratory grade.

### 2.3. Data Analysis

In the present study, the health risk of heavy metals contents in two *Pleurotus* spp. was analyzed using the target hazard quotient (THQ) method [26,27]. The cumulative health risk of heavy metals in child and adult human groups was assessed using the THQ index, as given in Equation (1):

(1) $$\mathrm{THQ}=10^{-3}\times \frac{Ef\times Ed\times Ir\times HMc}{Bw\times Cp\times Rd}$$

Moreover, the THQ sum of six heavy metals was used to present the cumulative health risk index (HRI) value. Following Equation (2) were used to calculate HRI:

(2) $$\mathrm{HRI}=\sum^{\;}{\mathrm{THQ}}_{\mathrm{Cd}+\mathrm{Cr}+\mathrm{Cu}+\mathrm{Fe}+\mathrm{Mn}+\mathrm{Zn}}$$

where, *Ef* is exposure frequency of heavy metals, *Ed* is exposure duration (365 days), *Ir* is the mushroom ingestion rate, *HMc* is the heavy-metal concentration in the mushroom sample (mg/kg), *Bw* is the average body weight (70 kg for adult and 16 kg for child), *Cp* is the consumption period (25,550 days for adult and 5840 days for child), *Rd* is the reference doses (Cd: 5.0 × 10^−4^; Cr: 3.0 × 10^−3^; Cu: 4.2 × 10^−2^; Fe: 7.0 × 10^−1^; Mn: 1.4 × 10^−2^, and Zn: 3.0 × 10^−1^) [28], and 10^−3^ is the conversion factor, respectively. Moreover, the data were analyzed using principal component analysis (PCA) and hierarchical clustering (CL) to derive the relationship between heavy-metal concentration in *Pleurotus* spp. and their sampling locations (correlation matrix). CL is used to create clusters that have a predetermined ordering from top to bottom. Based on their similarity, nodes are compared with each other where larger groups are built by joining groups of nodes. The data were tested using a one-way analysis of variance (ANOVA) and a Tukey’s post hoc test. The level of statistical significance was adjusted as the probability (*p*) < 0.05.

### 2.4. Software and Tools

Microsoft Excel 2019 (Microsoft, Redmond, WA, USA) and OriginPro 2022b (OriginLab, Northampton, MA, USA) software packages were used for the data analysis and visualization.

## 3. Results and Discussion

### 3.1. Heavy Metal Levels in Collected Pleurotus spp.

The results of heavy-metals concentration in *Pleurotus* spp. collected from different locations of Rajaji National Park are shown in Table 2. One-way ANOVA revealed significantly varying (*p* < 0.05) heavy-metal contents among each species between selected locations. Both *Pleurotus* spp. showed the following mean heavy-metals compositional order: Fe > Zn > Cu > Mn > Cr > Cd. The contents of Cd ranged between 0.03 and 0.16, and between 0.05 and 0.11 for *P. ostreatus* and *P. djamor*, respectively, in all studied locations. Although, the mean Cd contents found in *P. ostreatus* and *P. djamor* were 0.10 and 0.08 mg/kg, respectively, noting no risk to human health; numerous locations (except Chilla Forest Colony, Chandi Devi Forest, Sureshwari Devi, and Mansa Devi Forest) outlined Cd contents in *P. ostreatus*, approaching the safe limit set by Indian Standards (0.10 mg/kg) [28]. Conversely, *P. djamor* showed a value exceeding the safe limit only in Bijnor Canal Road (0.11 mg/kg). Moreover, the skewness test of heavy-metal contents in *Pleurotus* spp. showed both positive (0.05 to 1.76) and negative (–0.02 to –1.88) values, depicting random symmetric distribution throughout the sampling locations. Also, the Kurtosis tests showed both positive (0.23 to 1.07) and negative (–0.16 to –0.85) values, indicating that the data tend to have fewer outliers.

In a previous study, *P. ostreatus* was acknowledged to have a high affinity to bioaccumulate Cd, especially when grown widely [30]; this is concordant with the findings of the current study. Other wild mushroom species (*Trichloma spp.*) denoted high Cd contents in mushroom caps and stipes, with the former higher than the latter (Cd in caps > C in stipes) [9]. In Cd-contaminated locations, mass residential colonies were associated with mass tourism for which numerous rest houses are thriving for the last decades releasing their wastes to the nearby areas. Moreover, several famous temples attracting locals for praying and religious movements could be a source of contamination due to significant amounts of liberated wastes [31]. Furthermore, Bijnor Canal Road is famous for its mass transport between trees without any preliminary landscape planning. Tourist stations have been considered as main sources of organic and inorganic pollution, including heavy metals [32]. Similarly, toxic heavy-metal contents tend to accumulate in forest soils in the vicinity of roadways [33].

Annually, the Rajaji National Park witnesses spring water overflowing on the surface; this type of water often transports debris enclosing contaminants and other pollutants according to an earlier report from Penn State University [34], which may explain the unsafe Cd content found in several samples. Cd is considered one of the most dangerous metals found in mushrooms [35] and can seriously affect kidneys, and the human respiratory and reproductive systems [36,37]. Cr, Cu, Fe, and Zn contents found in *P. ostreatus* and *P. djamor* samples collected from all locations were far below the safe limits; [28,29] being in the following respective ranges: 0.57–1.14 mg/kg and 0.38–0.95 mg/kg, 13.52–19.20 mg/kg and 12.08–16.26 mg/kg, 21.88–35.66 mg/kg and 22.92–32.07 mg/kg, 7.80–12.05 mg/kg and 7.18–11.32 mg/kg, 13.56–22.10 mg/kg and 12.02–19.58 mg/kg. These metals are essential for human body nutrition and development when found in low contents. Skewness and Kurtosis values were all between –2 and +2 which shows an acceptable asymmetry and a normal univariate distribution [38].

### 3.2. Multivariate Analysis Results

**Figure 2:**
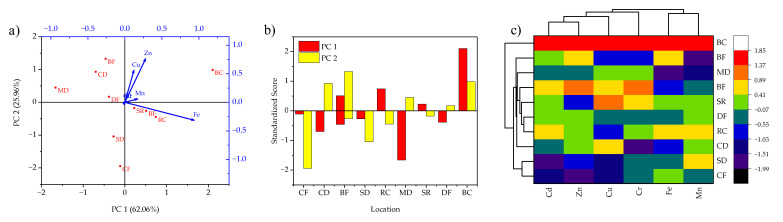
(**a**) Axial biplot; (**b**) PCA-standardized scores; and (**c**) HCA dendrogram with heatmap for heavy metal contents in *P. ostreatus* collected from different locations of Rajaji National Park, Haridwar, India.

The multivariate analysis provides better insights into the understanding of randomly distributed data sets [23]. In this study, principal component and hierarchal cluster analyses were used to derive the relationship and similarities between the heavy metal contents in two *Pleurotus* spp. and their sampling locations in and around Rajaji National Park, Haridwar, India. Results revealed that by using the PCA tool, the randomly distributed data was orthogonally transformed onto two principal components namely PC1 and PC2 for both *Pleurotus* spp. The standardized score plot given in Figure 2a showed the relative dominance of different PCs within the sampling locations. In particular, the PCs of *P. ostreatus* had eigenvalues of 16.50 and 6.90 covering the variances of 62.06 and 25.96%, respectively Figure 2b. For *P. ostreatus*, the axial vector length of Fe was highly correlated for Rishikesh Canal Road, Bheemgoda Barrage Forest, and Sapt Rishi Ghat, sampling sites while Zn and Cu dominated the Bijnor Canal Road location. Herein, the Mansa Devi Forest location showed the lowest heavy-metal contents For *P. ostreatus*, which suggested the safest location for *P. ostreatus* cultivation. Figure 2c shows the hierarchal cluster dendrogram with heatmap for heavy-metal contents in *P. ostreatus* collected from different locations of Rajaji National Park, Haridwar, India. Hierarchal cluster analysis showed that Mn–Fe and Cd–Zn had the highest similarities in terms of analyzed heavy-metals concentration in *P. ostreatus* mushroom. Also, similarities were identified between different sampling locations such as Sureshwari Devi Forest–Chilla Forest Colony and Devpura Forest Colony–Rishikesh Canal Road, respectively.

**Figure 3:**
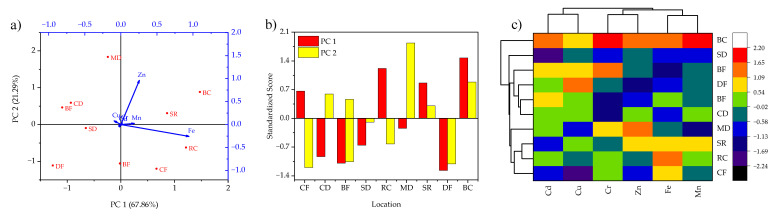
(**a**) Axial biplot; (**b**) PCA-standardized scores and (**c**) HCA dendrogram with heatmap for heavy metal contents in *P. djamor* collected from different locations of Rajaji National Park, Haridwar, India.

On the other hand, the extracted eigenvalues of PC1 and PC2 for heavy metal contents in *P. djamor* samples were observed as 15.30 and 4.80 depicting 67.86 and 21.29% variance, respectively. As given in Figure 3a, the vector length showed the highest concentration of Fe at Bijnor Canal Road, Rishikesh Canal Road, Sapt Rishi Ghat, and Chilla Forest Colony. Moreover, Zn was observed highest at Bijnor Canal Road, while other heavy metals showed relatively similar vector lengths at different sampling locations. Figure 3b shows the distribution of PCs over different sampling locations and confirmed that values of some scores positively dominated PC1 and PC2. Similarly, Figure 3c shows that Mn–Fe, Zn–Cr, and Cd–Cu lie within the same clusters due to their similarities in terms of analyzed heavy metal concentrations in *P. djamor* samples. However, the similarities between sampling locations were not effectively established due to random distribution except for Sapt Rishi Ghat–Rishikesh Canal Road and Bheemgoda Barrage Forest–Chandi Devi Forest.

Multivariate statistical analyses are one of the most effective methods for generalizing randomly distributed large data sets. In a recent study by Kumar et al. [12], the contents of heavy metals in *Agaricus bisporus* mushroom collected from different locations in Uttarakhand State, India were analyzed using geospatial and multivariate statistical tools i.e., principal component analysis and cluster analysis. They found that both PCA and HCA helped identify the districts which had the highest heavy-metal contamination across the state. Similarly, Sarikurkcu et al. [39] also analyzed the heavy-metal concentrations in eight selected *Russula* spp. from Turkey. They found that the variability of essential and non-essential heavy metals was successfully depicted using PCA and HCA. Moreover, Barea-Sepúlveda et al. [40] also utilized PCA and HCA tools to understand the variabilities of toxic heavy metals occurrence in *Macrolepiota procera* mushrooms collected from different locations in Spain and Morocco. Thus, these reports suggested that PCA and HCA can be useful for understanding the heavy metal variabilities in mushroom samples.

### 3.3. Results of Health Risk Studies

**Figure 4:**
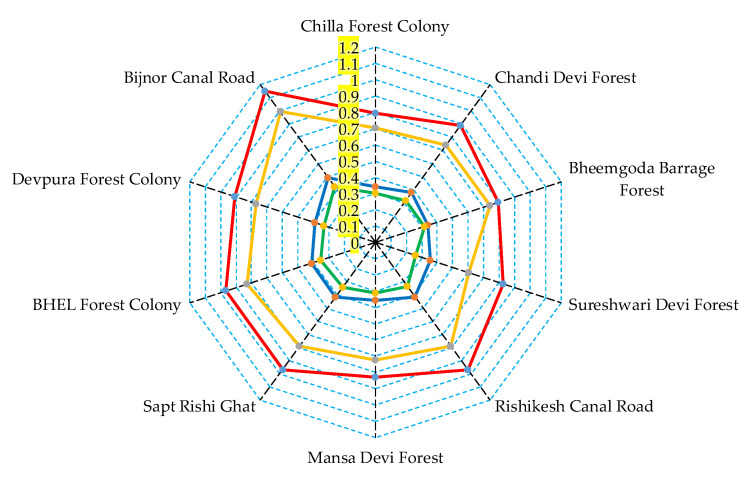
Spider-web diagram showing the health risk index (HRI) values of heavy metal contents in two oyster mushroom spp. (PO: *P. ostreatus* and PD: *P. djamor*) collected from different locations of Rajaji National Park, Haridwar, India.

As wild mushroom consumption has increased in numerous regions of the world, especially in developing countries, the need to investigate any possible health risks has become indispensable. Therefore, this can be predicted by studying the target hazard quotient (THQ) and health risk index (HRI) in adult and child groups for a better insight into any possible toxicity on human health [6]. Here, Table 3 summarizes the values of adult and child THQs for each heavy metal found in *P. ostreatus* and *P. djamor*, as well as their resultant HRIs. It was revealed that child THQs were always higher than the adult and that THQs of *P. ostreatus* were higher compared to those of *P. djamor*. A similar observation was recently acknowledged by Kumar et al. [12] regarding *Agaricus bisporus* mushrooms collected from Uttarakhand state markets. The study of HRIs on *Tricholoma spp.* revealed that they were higher in mushroom caps than in mushroom stipes [9]. Conversely, the current study evaluated THQs and HRIs of whole *Pleurotus spp.* mushrooms; therefore, further studies should be put into action to detect any possible potential risk associated with a specific part of the edible mushrooms. The average child HRIs of *P. ostreatus* and *P. djamor* was 2.33-fold higher than the average adult HRIs in the same species. Moreover, child and adult HRIs were higher by 1.1–1.4-fold in *P. ostreatus* than in *P. djamor*, as shown in Figure 4. All HRIs were < 1 outlining a safe consumption without potential health risk [41], except child HRI of *P. ostreatus*, collected from Bijnor Canal Road (1.151), which was mostly attributed to the high Cd content.

This region is currently heavily affected by the increasing activities of neighboring mines besides the uprising water canal pollution, although a restoration process took place in 2014. Accordingly, children’s intake of *P. ostreatus* from Bijnor Canal Road should be avoided as they may pose a serious effect on the development of their central nervous system leading to learning disabilities besides possible cancerogenic impacts [42]. For *P. ostreatus*, child HRIs’ in studied locations followed this decreasing order: Bijnor Canal Road > Rishikesh Canal Roa –Sapt Rishi Gat > BHEL Forest Colony > Devpura Forest Colony > Chandi Devi Forest > Mansa Devi Forest > Sureshwari Devi Forest > Bheemgoda Barrage Forest > Chilla Forest Colony; whereas, adult HRIs’ decreasing order was as follows: Bijnor Canal Road > Rishikesh Canal Road–Sapt Rishi Gat > BHEL Forest Colony > Devpura Forest Colony > Chandi Devi Forest > Mansa Devi Forest > Sureshwari Devi Forest > Bheemgoda Barrage Forest–Chilla Forest Colony.

For *P. djamor*, the following child HRIs trend was noted: Bijnor Canal Road > BHEL Forest Colony > Sapt Rishi Gat > Rishikesh Canal Road > Devpura Forest Colony > Bheemgoda Barrage Forest > Chandi Devi Forest > Mansa Devi Forest > Chilla Forest Colony > Sureshwari Devi Forest. Whereas, adult HRIs had the following decreasing order: Bijnor Canal Road > BHEL Forest Colony > Sapt Rishi Gat > Rishikesh Canal Road > Devpura Forest Colony > Chandi Devi Forest–Bheemgoda Barrage Forest > Mansa Devi Forest > Chilla Forest Colony > Sureshwari Devi Forest. The obtained findings simulate that HRI values were not only species-related, but also soil-related (mainly Cd contamination). Other recent studies outlined the strong inter-relationship between soil mercury (Hg) content and HRI values in wild edible mushrooms [40,43,44]. Thus, the THQ approach in the current study was helpful to estimate potential health risks associated with consumption of heavy metal contaminated *Pleurotus* spp.

## 4. Conclusions

This study concluded that two oyster mushrooms (*P. ostreatus* and *P. djamor*) collected from the Rajaji National Park, Haridwar, India showed the occurrence of selected heavy metals (Cd, Cu, Cr, Fe, Mn, and Zn). Comparatively, the contents of six heavy metals were found at maximum in *P. ostreatus* followed by *P. djamor*. However, the concentration varied significantly (*p* < 0.05) based on the sampling locations. Overall, the decreasing order of mean heavy-metal concentration in both *Pleurotus* spp. was Fe > Zn > Cu > Mn > Cr > Cd. The findings revealed that the samples collected from locations close to the residential, industrial areas, and transportation activities showed relatively higher heavy-metal contents as compared to those collected from the deep forest areas as indicated by the multivariate analyses. Similarly, the health risk index studies also indicated safe consumption of *Pleurotus* spp. collected from all locations except for those near heavy traffic and industrial areas. Considering the importance of wild edible mushrooms in human nutrition, proper measures should be taken in terms of heavy-metal contamination and associated health risks. Further studies on the biomonitoring of heavy metals in other edible mushroom species are highly recommended.

## Figures and Tables

**Table 1:** Description of the *Pleurotus* spp. sampling locations in Rajaji National Park, Haridwar, India.

Site Name	Code	Samples Collected in Two Years	Latitude (N) ^	Longitude (E) ^	Elevation (m)
Chilla Forest Colony	CF	*P. ostreatus* (*n* = 5); *P. djamor* (*n* = 4)	29°57′46.57″	78°11′41.59″	296
Chandi Devi Forest	CD	*P. ostreatus* (*n* = 5); *P. djamor* (*n* = 5)	29°56′10.38″	78°10′56.72″	517
Bheemgoda Barrage Forest	BF	*P. ostreatus* (*n* = 4); *P. djamor* (*n* = 5)	29°57′20.73″	78°11′10.16″	333
Sureshwari Devi Forest	SD	*P. ostreatus* (*n* = 5); *P. djamor* (*n* = 5)	29°58′70.49″	78° 6′26.45″	315
Rishikesh Canal Road	RC	*P. ostreatus* (*n* = 5); *P. djamor* (*n* = 5)	29°58′58.16″	78°14′20.12″	328
Mansa Devi Forest	MD	*P. ostreatus* (*n* = 5); *P. djamor* (*n* = 5)	29°57′24.01″	78° 9′56.09″	440
Sapt Rishi Ghat	SR	*P. ostreatus* (*n* = 5); *P. djamor* (*n* = 2)	29°58′40.43″	78°11′18.52″	299
BHEL Forest Colony	BF	*P. ostreatus* (*n* = 5); *P. djamor* (*n* = 4)	29°57′9.92″	78° 6′22.65″	304
Devpura Forest Colony	DF	*P. ostreatus* (*n* = 5); *P. djamor* (*n* = 2)	29°56′25.96″	78° 8′14.40″	322
Bijnor Canal Road	BC	*P. ostreatus* (*n* = 5); *P. djamor* (*n* = 2)	29°55′90.43″	78°10′27.73″	286

^: Coordinates and referencing system (CRS) is rendered as EPSG:4326–WGS 84 projection; Source: Google Earth Pro.

**Table 2:** Heavy-metal concentration (mean ± SD; *n* = 2 to 5) in two *Pleurotus* spp. collected from different locations of Rajaji National Park, Haridwar, India.

Sampling Site	*Pleurotus* spp.	Heavy-Metal Concentration (mg/kg Dry Weight)					
		Cd	Cr	Cu	Fe	Mn	Zn
Chilla Forest Colony	*P. ostreatus*	0.06 ± 0.02 ab	0.81 ± 0.14 b	14.10 ± 1.20 b	29.76 ± 1.53 c	9.33 ± 0.20 c	13.56 ± 0.62 a
	*P. djamor*	0.05 ± 0.01 b	0.73 ± 0.05 b	12.08 ± 0.08 a	30.20 ± 0.86 c	8.14 ± 0.15 b	14.09 ± 0.40 a
Chandi Devi Forest	*P. ostreatus*	0.10 ± 0.03 bc	0.57 ± 0.07 a	17.90 ± 1.04 cd	25.01 ± 3.17 ab	10.36 ± 1.10 c	18.64 ± 1.56 c
	*P. djamor*	0.07 ± 0.02 ab	0.42 ± 0.08 a	15.05 ± 0.49 bc	23.19 ± 2.02 a	8.99 ± 0.72 b	16.03 ± 1.10 b
Bheemgoda Barrage Forest	*P. ostreatus*	0.11 ± 0.03 c	0.75 ± 0.09 b	14.43 ± 0.32 b	30.86 ± 1.38 c	7.80 ± 0.43	19.70 ± 0.98 c
	*P. djamor*	0.09 ± 0.01 c	0.38 ± 0.02 a	15.09 ± 0.75 b	27.74 ± 0.84 bc	8.64 ± 0.27 b	13.50 ± 2.15 a
Sureshwari Devi Forest	*P. ostreatus*	0.03 ± 0.01 a	0.84 ± 0.10 bc	13.52 ± 0.61 ab	28.04 ± 2.03 c	10.93 ± 1.09 c	16.27 ± 1.53 b
	*P. djamor*	*Bdl*	0.45 ± 0.15 a	14.19 ± 0.87 b	24.82 ± 4.20 ab	7.55 ± 0.14 a	15.01 ± 1.07 b
Rishikesh Canal Road	*P. ostreatus*	0.13 ± 0.02 c	0.92 ± 0.03 c	15.06 ± 0.16 bc	31.56 ± 1.64 c	10.70 ± 0.81 c	18.65 ± 1.94 c
	*P. djamor*	0.08 ± 0.02 ab	0.64 ± 0.07 b	14.15 ± 0.90 b	32.07 ± 1.98 c	9.07 ± 0.29 c	15.71 ± 0.62 b
Mansa Devi Forest	*P. ostreatus*	0.09 ± 0.01 c	0.89 ± 0.08 c	16.21 ± 0.19 c	21.88 ± 0.43 a	8.31 ± 0.37 b	17.10 ± 0.78 b
	*P. djamor*	0.07 ± 0.01 b	0.77 ± 0.04 c	13.62 ± 1.35 a	25.29 ± 1.05 ab	7.18 ± 0.11 a	19.58 ± 0.25 c
Sapt Rishi Ghat	*P. ostreatus*	0.11 ± 0.03 bc	0.96 ± 0.05 c	18.40 ± 0.46 d	29.67 ± 0.57 c	10.11 ± 0.22 c	16.39 ± 2.38 b
	*P. djamor*	0.05 ± 0.01 b	0.62 ± 0.12 b	15.10 ± 1.10 b	30.16 ± 1.32 c	9.75 ± 0.45 c	17.22 ± 0.70 b
BHEL Forest Colony	*P. ostreatus*	0.13 ± 0.02 cd	1.04 ± 0.02 d	17.21 ± 0.84 cd	25.64 ± 1.88 b	9.42 ± 0.60 c	20.84 ± 0.58 c
	*P. djamor*	0.10 ± 0.03 c	0.85 ± 0.03 c	16.12 ± 1.32 c	22.92 ± 3.05 a	8.09 ± 0.44 a	15.58 ± 1.39 b
Devpura Forest Colony	*P. ostreatus*	0.11 ± 0.02 c	0.79 ± 0.14 b	15.88 ± 0.78 b	26.78 ± 1.19 b	10.25 ± 0.97 c	18.24 ± 0.66 c
	*P. djamor*	0.08 ± 0.01 c	0.57 ± 0.09 b	16.26 ± 0.35 c	23.08 ± 3.60 a	8.58 ± 0.48 b	12.02 ± 2.04 a
Bijnor Canal Road	*P. ostreatus*	0.16 ± 0.02 d	1.14 ± 0.17 c	19.20 ± 0.40 d	35.66 ± 0.94 cd	12.05 ± 0.51 d	22.10 ± 0.71 d
	*P. djamor*	0.11 ± 0.04 c	0.95 ± 0.09 c	16.07 ± 0.69 c	32.02 ± 1.55 c	11.32 ± 0.35 bc	18.90 ± 1.20 c
Minimum	*P. ostreatus*	0.03	0.57	13.52	21.88	7.80	13.56
	*P. djamor*	0.05	0.38	12.08	22.92	7.18	12.02
Maximum	*P. ostreatus*	0.16	1.14	19.20	35.66	12.05	22.10
	*P. djamor*	0.11	0.95	16.26	32.07	11.32	19.58
Median	*P. ostreatus*	0.11	0.87	16.05	28.86	10.18	18.44
	*P. djamor*	0.08	0.63	15.07	26.52	8.61	15.65
Mean	*P. ostreatus*	0.10	0.87	16.19	28.49	9.93	18.15
	*P. djamor*	0.08	0.64	14.77	27.15	8.73	15.76
SD	*P. ostreatus*	0.04	0.16	1.94	3.89	1.26	2.46
	*P. djamor*	0.02	0.19	1.30	3.74	1.18	2.34
Skewness	*P. ostreatus*	−0.68	−0.16	0.17	0.13	−0.21	−0.23
	*P. djamor*	0.05	0.17	−0.85	0.18	1.07	0.22
Kurtosis	*P. ostreatus*	0.82	0.62	−1.31	0.31	−0.02	0.23
	*P. djamor*	−0.70	−0.97	0.62	−1.88	1.76	−0.35
Safe Limit [28,29]	*-*	0.10	2.30	40.00	425.00	30.00	50.00

The same letters (a–d) indicate no significant difference between the sampling location values at *p* < 0.05. *Bdl*: below detectable limits.

**Table 3:** Health risk index of heavy metal contents collected from different locations of Rajaji National Park, Haridwar, India.

Sampling Site	*Pleurotus* spp.	Target Hazard Quotient (THQ)											
		Cd		Cr		Cu		Fe		Mn		Zn	
		Child	Adult	Child	Adult	Child	Adult	Child	Adult	Child	Adult	Child	Adult
Chilla Forest Colony	*P. ostreatus*	0.065	0.028	0.141	0.060	0.182	0.078	0.023	0.010	0.360	0.154	0.024	0.010
	*P. djamor*	0.054	0.023	0.132	0.056	0.156	0.067	0.023	0.010	0.314	0.135	0.025	0.011
Chandi Devi Forest	*P. ostreatus*	0.108	0.046	0.099	0.042	0.230	0.099	0.019	0.008	0.400	0.171	0.034	0.014
	*P. djamor*	0.076	0.032	0.076	0.032	0.194	0.083	0.018	0.008	0.347	0.149	0.029	0.012
Bheemgoda Barrage Forest	*P. ostreatus*	0.119	0.051	0.130	0.056	0.186	0.080	0.024	0.010	0.301	0.129	0.036	0.015
	*P. djamor*	0.097	0.042	0.068	0.029	0.194	0.083	0.021	0.009	0.334	0.143	0.024	0.010
Sureshwari Devi Forest	*P. ostreatus*	0.032	0.014	0.146	0.063	0.174	0.075	0.022	0.009	0.422	0.181	0.029	0.013
	*P. djamor*	0.000	0.000	0.081	0.035	0.183	0.078	0.019	0.008	0.292	0.125	0.027	0.012
Rishikesh Canal Road	*P. ostreatus*	0.141	0.060	0.160	0.069	0.194	0.083	0.024	0.010	0.413	0.177	0.034	0.014
	*P. djamor*	0.087	0.037	0.115	0.049	0.182	0.078	0.025	0.011	0.350	0.150	0.028	0.012
Mansa Devi Forest	*P. ostreatus*	0.097	0.042	0.155	0.066	0.209	0.089	0.017	0.007	0.321	0.138	0.031	0.013
	*P. djamor*	0.076	0.032	0.139	0.059	0.175	0.075	0.020	0.008	0.277	0.119	0.035	0.015
Sapt Rishi Ghat	*P. ostreatus*	0.119	0.051	0.167	0.072	0.237	0.102	0.023	0.010	0.390	0.167	0.030	0.013
	*P. djamor*	0.054	0.023	0.112	0.048	0.194	0.083	0.023	0.010	0.377	0.161	0.031	0.013
BHEL Forest Colony	*P. ostreatus*	0.141	0.060	0.181	0.077	0.222	0.095	0.020	0.008	0.364	0.156	0.038	0.016
	*P. djamor*	0.108	0.046	0.153	0.066	0.208	0.089	0.018	0.008	0.312	0.134	0.028	0.012
Devpura Forest Colony	*P. ostreatus*	0.119	0.051	0.137	0.059	0.204	0.088	0.021	0.009	0.396	0.170	0.033	0.014
	*P. djamor*	0.087	0.037	0.103	0.044	0.209	0.090	0.018	0.008	0.331	0.142	0.022	0.009
Bijnor Canal Road	*P. ostreatus*	0.173	0.074	0.198	0.085	0.247	0.106	0.028	0.012	0.465	0.199	0.040	0.017
	*P. djamor*	0.119	0.051	0.171	0.073	0.207	0.089	0.025	0.011	0.437	0.187	0.034	0.015

## Data Availability

Not applicable.
